# The Driving Forces of Carbon Dioxide Equivalent Emissions Have Spatial Spillover Effects in Inner Mongolia

**DOI:** 10.3390/ijerph16101735

**Published:** 2019-05-16

**Authors:** Yannan Zhou, Jixia Huang, Mingxiang Huang, Yicheng Lin

**Affiliations:** 1 Key Laboratory for Silviculture and Conservation of Ministry of Education, Beijing Forestry University, Beijing 100083, China; zhouyn.17s@igsnrr.ac.cn (Y.Z.); minmengfann@hotmail.com (Y.L.); 2 Institute of Geographic Sciences and Natural Resources Research, Chinese Academy of Sciences, Beijing 100101, China; 3 Information Center of Ministry of Ecology and Environment, Beijing 100029, China

**Keywords:** county-level CO_2eq_ emissions, Inner Mongolia, spatial panel model, driving factors

## Abstract

To spatially analyze the effects of the major drivers on carbon dioxide equivalent (CO_2eq_) emissions in Inner Mongolia, a typical area with high CO_2eq_ emissions in China, this paper quantitatively investigates the factors that affect county-level CO_2eq_ emissions and the corresponding spatial mechanisms. Based on a spatial panel econometric model with related energy and economic data from 101 counties in Inner Mongolia between 2007 and 2012, four main results are obtained: (a) The CO_2eq_ emissions in Inner Mongolia rapidly increased at an average annual growth rate of 7.27% from 2007 to 2012, increasing from 287.69 million tons to 510.47 million tons. (b) The county-level CO_2eq_ emissions in Inner Mongolia increased, but the growth rate decreased annually. Additionally, CO_2eq_ emissions are highly heterogeneous in the region. (c) Geographic factors were the main cause of the spatial spillover effects related to county-level CO_2eq_ emissions. Specifically, the levels of urbanization and technological progress were conducive to CO_2eq_ emission reductions, and the economic growth and industrial structure had the opposite effect in Inner Mongolian counties. (d) Technological progress had a significant spatial spillover effect in Inner Mongolian counties, and the effects of other factors were not significant. Implementing relevant strategies that focus on the inter-county interactions among the driving forces of CO_2eq_ emissions could promote energy savings and emission reductions in Inner Mongolia.

## 1. Introduction

Global carbon dioxide equivalent (CO_2eq_) emissions from fossil energy combustion and industrial processes rapidly increased at an average annual rate of 1.8% from 2005 to 2017 and were projected to reach an all-time high in 2018 [1]. With such a high-speed carbon emissions growth model, energy savings and emission reductions have become increasingly imperative [2,3,4,5]. China, as a major energy-consuming and carbon-emitting country, has increasingly attracted global research attention and has overtaken the United States as the world’s largest carbon emitter since 2006 [6,7]. Specifically, CO_2eq_ emissions in China account for 27% of global emissions from fossil combustion and industrial processes [1]. With an emphasis placed on energy savings and emission reductions, the Chinese government has pledged to decrease its CO_2eq_ emissions intensity by 40–45% by 2020 compared to that in 2005 [8,9]. Therefore, low-carbon development must be urgently promoted in China [10].

Previous studies of CO_2eq_ can be divided into four categories: estimations and accountings of carbon emissions [11,12,13,14], the mechanisms of and factors that influence CO_2eq_ emissions [15,16,17,18,19,20], scenario analysis and predictions of CO_2eq_ emissions [21,22,23], and technology- and policy-based simulations of CO_2eq_ emission reductions [24,25]. In recent years, there has been increased interest in the factors that influence energy-related CO_2eq_ emissions [26]. The literature on the methods used to explore the factors that affect CO_2eq_ emissions can be broadly divided into two categories [27]. The first category includes decomposition methods, such as structural decomposition analysis (SDA), index decomposition analysis (IDA), and production theory decomposition analysis (PDA) [28]. The second category includes econometric analysis methods, such as panel models, the Stochastic Impacts by Regression on Population, Affluence, and Technology (STIRPAT) model, and spatial panel models [27].

The factors that influence CO_2eq_ emissions vary in different research methods and study areas. Many previous studies have employed decomposition methods, and the logarithmic mean divisia index (LMDI) model is the most commonly used in IDA. Using this method, de Freitas and Kaneko [29] found that reducing the CO_2eq_ intensity and modifying the energy structure were the key methods used to reduce Brazilian emissions from 2004 to 2009. In South Korea, Jung et al. [30] found that increases in CO_2eq_ emissions were mainly due to production effects in eco-industrial parks (EIPs) and the surrounding regions. In China, Li et al. [31] concluded that the factors that influence emissions differ at the provincial level but that the economy is the most important contributor. In a group of 18 developed economies, Le Quéré et al. [32] demonstrated that the reduction in CO_2eq_ emissions could be explained by the displacement of fossil fuels by renewable energy and decreases in energy use from 2005–2015. Additionally, other studies have identified relevant impact factors based on econometric analysis. Al-mulali and Binti Che Sab [33] studied 30 sub-Saharan African countries and found that energy consumption played a strong role in promoting CO_2eq_ emissions and economic growth. Saboori and Sulaiman [34] also confirmed this conclusion. Burnett et al. [35] found that the effects of positive economic spillover and negative price spillover on state-level carbon emissions are important in the United States. Wang et al. [36] researched the variable effects of the urbanization rate, industrial structure, energy consumption structure, and foreign direct investment in eight regions, as well as the county-level carbon intensity. Zhang and Xu [37] concluded that both land urbanization and land finance significantly influence carbon emissions and that the high speed of land urbanization has reduced Chinese carbon emissions. Additionally, Zhou and Liu [38] suggested that income is the key factor linked to increased carbon emissions in China.

Previous studies of CO_2eq_ emissions have mainly focused on the national [11,33,39], regional [5,40,41], and city levels [35,42,43]. However, there is a relative lack of research at the microscale of the county level. Inner Mongolia is the largest province in China. The province consists of 101 counties that encompass an area of 1.18 million square kilometers and account for nearly one-eighth of the territory of China. Inner Mongolia is an important Chinese energy production base, an important gateway to the west, and a ‘Belt and Road’ core area of the Chinese, Mongolian, and Russian economic zone. Therefore, the province is currently in an important stage of industrialization. The economy has rapidly grown, especially from 2002–2009, at a speed of 18.45% (The data are from the Public Service Platform of the Inner Mongolia Autonomous Region Bureau of Statistics (http://www.nmgtj.gov.cn/acmrdatashownmgpub/index.htm)) annually. Rapid economic growth in parallel with industrialization and urbanization has resulted in contradictions between economic development and environmental protection [9]. In 2015, Inner Mongolia was the fourth largest emitting province in China after Shandong, Jiangsu, and Hebei Provinces, and CO_2eq_ emissions totaled 584.7 million tons [44]. However, the population of Inner Mongolia is much lower than those of the other three top carbon-emitting provinces, and the economy is less developed. The phenomena of large-scale development, high-intensity exploitation, and high fossil energy consumption in Inner Mongolia are particularly prominent. Such high carbon development has further deteriorated the environment and led to decelerated economic development and increased CO_2eq_ emissions [45]. According to previous research, the climate warming rate in Inner Mongolia is over double that globally [46]. Therefore, studying the energy-related CO_2eq_ emissions in Inner Mongolia and the key factors related to emission reductions at the county level is significant for the coordinated development of society, the economy, energy, and the environment in Inner Mongolia.

In Inner Mongolia, each county can be regarded as a spatial unit that is often significantly interdependent on other counties, rather than independent or randomly related [47]. This significant interdependence is called spatial dependence. Most previous studies regarded spatial units as independent and homogeneous individuals, ignoring the spatial relations among adjacent units [9,48]. Therefore, we employ a spatial panel econometric model that considers spatial heterogeneity and dependence to illustrate the spatiotemporal mechanisms of the driving forces of county-level CO_2eq_ emissions. By accounting for coupled spatial and temporal effects, a spatial panel econometric model can yield a spatial regression model of the established factors that is highly consistent with reality [49]. Therefore, this approach meticulously yields the spatiotemporal evolution and driving mechanisms of CO_2eq_ emissions in Inner Mongolian counties.

Above all, this paper explores the spatiotemporal distribution of CO_2eq_ emissions in Inner Mongolian counties by spatial visualization. Then, the spatial panel econometric model is applied to identify the core driving forces of county-level CO_2eq_ emissions in Inner Mongolia and the corresponding spatial spillover effects. The results can be used in provincial-level and county-level emission reduction tasks and provide a reference for low-carbon development in provinces with high carbon emissions in the western region of China.

## 2. Data and Methods

### 2.1. Data

**Figure 1:**
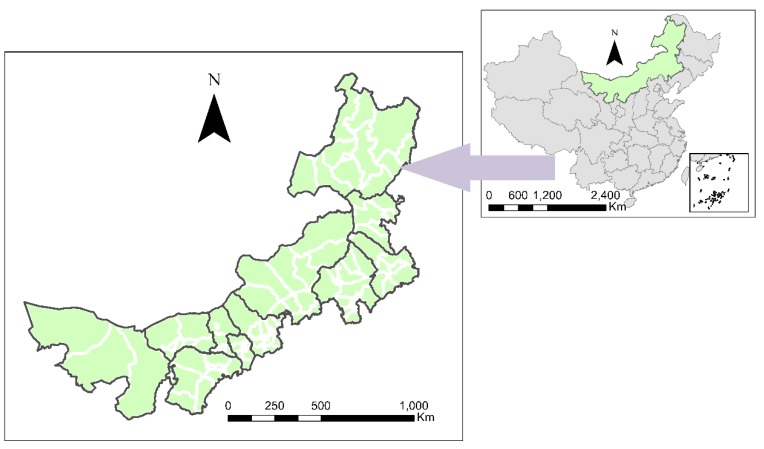
The study area.

This study considered 101 county-level administrative units in Inner Mongolia (Figure 1), and the driving forces of CO_2eq_ emissions changes were investigated from 2007 to 2012. The data employed in this paper (Table 1) were primarily taken from the *Inner Mongolia Almanac* [50], the *Inner Mongolia Statistical Yearbook* [51], and the statistical yearbooks of all counties in Inner Mongolia. Specifically, the socioeconomic data were taken from the *National Economic and Social Development Annual* reports. In addition, the energy-related data were from energy and environmental investigations and the literature. It should be noted that the CO_2eq_ emissions data employed in this paper refer to the CO_2eq_ emissions produced by the industrial sector in all Inner Mongolian counties. These values were calculated via the energy coefficient method. The industrial sector classification in this study follows that of the *Industrial Classification for National Economic Activities (GB/T 4754-2011)* issued by the *National Bureau of Statistics* and covers 39 industries under the second-level classification. In this paper, CO_2eq_ emissions are considered an explained variable, and the explanatory variables are the urbanization rate (Urban), per capita GDP (PGDP), technical progress (TP), industrial structure (IS), and the output of the construction industry (VC).

### 2.2. Method

#### 2.2.1. Eco-Spatial Weighting Matrix

The traditional geographical weighting matrix follows the *ROOK Adjacent Judgement Principle* [9]. However, different spatial interactions exist in different areas. Less developed areas have little impact on developed areas, but the impact on developed areas is the opposite, i.e., a more powerful spatial impact is often observed [52]. An eco-spatial weighting matrix reflects the regional economic level by calculating the average value of the real GDP per capita in a region based on the population in all regions. In this approach, the eco-spatial weighting matrix can be used to explore the specific radiation effects of different economic levels. The corresponding formula can be written as follows:

(1) $$\left\{ \begin{array}{l} W=w*diag(\frac{\bar{y_{1}}}{\bar{y}},\frac{\bar{y_{2}}}{\bar{y}},\cdots ,\frac{\bar{y_{n}}}{\bar{y}})\\ \bar{y_{i}}=\frac{1}{t_{1}-t_{0}+1}\sum_{t=t_{0}}^{t_{i}}y_{it}\\ \bar{y}=\frac{1}{n(t_{1}-t_{0}+1)}\sum_{t=t_{0}}^{t_{i}}\sum_{i=1}^{n}y_{it} \end{array}\right.$$

where *W* and *w* denote the eco-spatial and geographical weight matrixes, respectively; *y_it_* denotes the real per capita GDP in the *i_th_* area during the *t_th_* year; and *y_i_* and $\bar{y}$ represent the average real GDP per capita in the *i_th_* area and in all areas from *t*_0_ to *t*_1_, respectively.

#### 2.2.2. Types of Models

Most standard methods in spatial analysis begin with a non-spatial linear regression model to determine whether the model can be extended with spatial interaction effects [53]. According to different “interactive spatial effects”, spatial panel models can be classified into four types: ordinary least squares (OLS), spatial autoregressive (SAR), the spatial error model (SEM), and the spatial Durbin model (SDM) [54].

Traditional econometric models that do not generally include spatial interactive effects may lead to incorrect results. Therefore, this study explored spatial panel modeling, including spatial and temporal effects, to determine the spillover effects of different variables. To select a more realistic spatial regression model and analyze the factors that influence CO_2eq_ emissions in Inner Mongolia, statistical tests of different spatial panel models were conducted. The test methods were as follows.

First, considering the residual tests of the non-spatial models, this study performed exchange tests with traditional Lagrange multipliers (LM) (includes the LMlag and LMerror tests) and robust Lagrange multipliers (R-LM) (includes the R-LMlag and R-LMerror tests) based on the SAR and SEM, respectively. Second, in terms of selecting between the SAR and SEM, we performed OLS estimation for the CO_2eq_ emissions in Inner Mongolia via a panel model without spatial interactions. The results are shown in Table 2, and they indicate that the panel model without spatial interactions is the most suitable model based on the results for both the null hypotheses and the geographical weighting matrix.

Additionally, the eco-spatial weighting matrix was rejected at a statistically significant level of 1% with LM and R-LM tests. The likelihood ratio (LR) tests, which were performed to assess the joint significance of the spatially fixed (estimated value = 2569.948, degrees of freedom = 101, *p* = 0.000) and temporally fixed (estimated value = 472.306, degrees of freedom = 6, *p* = 0.000) effects, indicated that the bi-directional fixed effects dominate over spatially and temporally fixed effects.

Finally, the Wald and LR tests were further considered to determine whether the SAR model or SEM is better. Table 2 shows that both results contradicted the null hypothesis at a statistically significant level of 5%. Additionally, the Hausman test result (estimated value = 198.928, degrees of freedom = 11, *p* = 0.000) indicated that the random effect model was rejected. Therefore, the SDM with bi-directionally fixed effects can most accurately describe the mechanisms of CO_2eq_ emissions in Inner Mongolian counties.

According to the model selection results, the SDM considers the interactive effects of endogenic, exogenic, and autocorrelated terms. Alternatively, the SAR with exogenous interactive effects and the SEM with autocorrelated perturbation error terms do not meet the required criteria. Therefore, SDM can effectively reveal the factors that influence the spatial interactions of CO_2eq_ emissions among counties. The CO_2eq_ emissions in Inner Mongolian counties can be expressed as follows:

(2) $$Y_{it}=\rho WY_{it}+\beta X_{it}+WX_{it}\alpha +\lambda_{t}+\mu_{i}+\varepsilon_{it},\;\varepsilon_{it}\sim N(0,\delta^{2}I_{N})$$

where *C_it_* represents area *i*’s CO_2eq_ emissions in period *t*; *X_it_* represents area *i*’s independent variable in period *t*; *β* represents the coefficient of the independent variable; *λ_t_* represents the temporal effects; *μ_i_* represents the spatial effects; *ε_it_* is the random error effect, which follows a normal distribution; and *α* represents the coefficient of the spatial lag (an explanatory variable). If *α* is statistically significant, there are spatial spillover effects associated with the dependent variables among neighboring spatial units.

#### 2.2.3. The Robustness Test

To perform a robustness test of the models, this paper employed spatial panel models based on two different weighting matrixes. One matrix was the eco-spatial weighting matrix, and the other was the geospatial weighting matrix.

## 3. Results

### 3.1. The Spatiotemporal Distribution of County-Level CO_2eq_ Emissions

**Figure 2:**
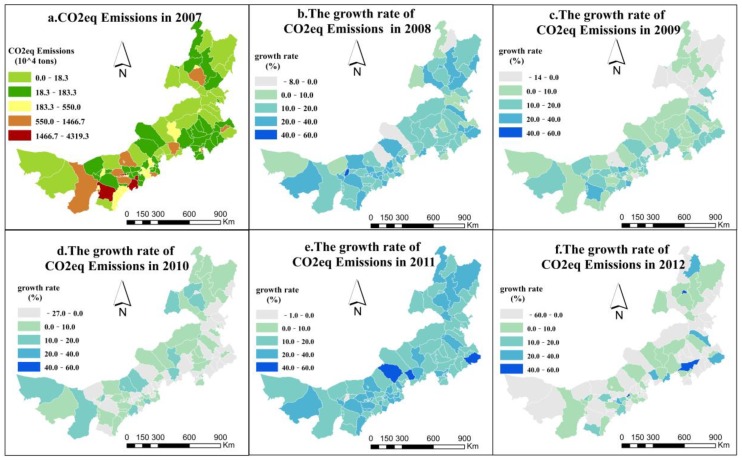
The spatiotemporal distribution of CO_2eq_ emissions and the annual growth rate in the counties of Inner Mongolia from 2007 to 2012. (Subfigure **a** representsCO_2eq_ emissions in 2007 and subfigure **b**–**f** represent growth rate of CO_2eq_ emissions compared to last year respectively. This type of visualization can show the temporal and spatial changes of CO_2eq_ emissions).

Inner Mongolia is a vast territory, and the CO_2eq_ emissions in this area are spatially heterogeneous. The CO_2eq_ emissions in the province increased rapidly from 2007 to 2012, with an average annual rate of increase of 7.27%. In 2012, CO_2eq_ emissions totaled 13,921.92 million tons and had increased by 6076.10 million tons comparing to the level in 2007. In 2012, from the minimum value of 0.0025 million tons in AGXIHB, Xilin Gol League (The Xilin Gol League is an alliance of the Inner Mongolia Autonomous Region located in North China, in the central part of the Inner Mongolia Autonomous Region. AGXIHB is a county affiliated with Xilin Gol, located in the southwestern part of the Xilin Gol grassland in Inner Mongolia. This area is an important new green industrial base, an important commercial base, and a trade logistics node and distribution center in the southern part of Xilin Gol.) to the maximum value of 1178.80 million tons in Otog, Erdos (Erdos is a prefecture-level city under the jurisdiction of Inner Mongolia located in southwestern Inner Mongolia. Otog is a county governed by Erdos and located in western Erdos.), the distribution of county-level CO_2eq_ emissions was spatially discrete but slightly agglomerated in some areas. Areas with high CO_2eq_ emissions were primarily located in southwestern and central Inner Mongolia, and eastern and northern Inner Mongolia emitted less CO_2eq_ emissions (Figure 2). Although the overall CO_2eq_ emissions in all counties in Inner Mongolia have increased annually, the growth rate has slowed (except in 2011). In addition, the number of counties with a negative growth rate has increased. In 2010 and 2012, there were more counties with decreased CO_2eq_ emissions compared to the situation in 2007. CO_2eq_ emissions decreased in 37 counties, primarily those located in southern Inner Mongolia, from 2009 to 2010. Two years later, the number of counties with reduced CO_2eq_ emissions increased to 52, including some in northern Inner Mongolia.

### 3.2. The Spatial Econometrics of the Driving Forces of County-Level CO_2eq_ Emissions

Based on a comparison of these two spatial weighting models, the fitting degree of the spatial panel data model based on economic weighting is relatively poor compared to that of the spatial panel model based on geographical weighting. Therefore, of the driving factors that affect the county-level CO_2eq_ emissions in Inner Mongolia, the impact of economic disparity is relatively less important than that of the geographical distance. Consequently, the SDM with spatiotemporal fixed effects based on the geospatial weighting matrix in Table 1 is more suited to investigating the driving mechanisms of county-level CO_2eq_ emissions in Inner Mongolian. It is worth noting that the model applied in this paper is robust and yields reliable results, as demonstrated by the traditional goodness-of-fit R^2^, the correct R^2^ excluding fixed effects, and the relation coefficient results.

In the geospatial weighting matrix, *Urban*, *PDGP,* and *TP* pass the 1% statistical significance test, and *IS* passes the 5% significance test. Therefore, *Urban*, *PDGP*, *TP*, and *IS* are considered the primary factors that influence the spatiotemporal pattern of CO_2eq_ emissions, and *VC* has no significant effect on the distribution in Inner Mongolian counties.

The urbanization rate is significantly negatively correlated with the CO_2eq_ emissions in Inner Mongolian counties during the sample period. According to Table 3 (column 6), the elasticity coefficient of *LnUrban* for CO_2eq_ emissions is −0.145 with a significance level of 1%, and the elastic hysteresis coefficient is 0.047 with a significance level of 10%. In this case, the direct effect is significant, and the indirect effect is not, implying that if one county increases its own urbanization rate by 1%, the county-level CO_2eq_ emissions would decrease by approximately 0.143% in that county and increase by 0.021% in neighboring areas. Thus, urbanization can promote local energy savings and emission reductions. However, the influence of urbanization is not obvious in neighboring areas.

The GDP per capita is related to CO_2eq_ emissions growth in local counties and has less of an effect on neighboring areas in Inner Mongolia. According to Table 3, the elasticity coefficient of *LnPGDP* is 0.365, with a significance level of 1%. In addition, the elastic hysteresis coefficient fails the significance test, indicating that spillover effects are not significant. According to the direct and indirect effects in Table 3 (columns 7 and 8), if the *PGDP* changes positively in local counties by 1%, the CO_2eq_ emissions in the county will significantly increase by 0.366%, and emissions in neighboring area will slightly and non-significantly increase by 0.063%.

Technological progress has had a negative impact on Inner Mongolian CO_2eq_ emissions. According to Table 3, the elasticity coefficient of *LnTP* for CO_2eq_ emissions is −0.807, with a significance level of 1%, far outweighing other driving factors. Thus, *LnTP* is considered the key factor associated with CO_2eq_ emissions in Inner Mongolian counties. The elastic hysteresis coefficient is 0.239 with a significance level of 1%. According to the direct and indirect effects in Table 3 (columns 7 and 8), if one county increases its own technology level by 1%, an approximate decrease of 0.807% in CO_2eq_ emissions will occur in the local area, and a significant increase of 0.100% will occur in neighboring areas. This result indicates that energy savings and emission reductions can result from technological progress. In addition, although technology generally increases CO_2eq_ emissions in neighboring areas, the influence is still weak.

Increasing the proportion of secondary industry in the industrial structure has a significantly positive influence on the county-level CO_2eq_ emissions in Inner Mongolia. According to Table 3, the elasticity coefficient of *LnIS* is 0.013 with a significance level of 5%, and the elastic hysteresis coefficient is −0.018, failing the significance test. As Table 3 (columns 7 and 8) shows, each 1% positive increase in secondary industry will lead to a significant 0.012% increase in CO_2eq_ emissions in the local area. Additionally, the spillover effects of the industrial structure are not significant, with a small negative impact of 0.019% in the neighboring areas.

The construction industry in Inner Mongolia has a negligible impact on county-level CO_2eq_ emissions. Table 3 shows that the elasticity coefficient, elastic hysteresis coefficient, direct effect, indirect effect, and gross effect results for *LnVC* are all not significant.

The CO_2eq_ emissions in all counties are influenced not only by the related local factors but also by CO_2eq_ emissions in neighboring areas. Table 3 indicates that the elastic hysteresis coefficient of *LnCE* is 0.191 with a significance level of 1%. Therefore, *LnCE* is associated with significant spatial spillover effects involving CO_2eq_ emissions in Inner Mongolian counties.

## 4. Discussion

Some scholars have suggested that with economic growth and an increase in per capita income, the environment will undergo an inverted U-shaped transformation process with initial deterioration and gradual improvement [55,56,57,58,59]. However, this study reveals that this transformation does not exist in Inner Mongolia. The effect of economic growth on carbon emissions was observed to be in the early stage of the inverted U-shaped curve; thus, economic growth has led to increased carbon emissions. Given that clean and regenerative models of production are not in place in Inner Mongolia, the economic growth pattern in Inner Mongolia is characterized as “extensive” and “high energy consumption”. These findings explain why the direct promoting effect of economic growth on county-level CO_2eq_ emissions is still the key driving factor and why spillover effects are not negligible. This relation primarily results from a relatively low level of development. Additionally, the energy production and technological utilization levels are relatively poor in Inner Mongolia.

As a province with high energy consumption, economic development in Inner Mongolia is backed by large quantities of “cheap” energy. Coal-based energy consumption structures result in rigid energy consumption demands in terms of economic development, inevitably contributing to a dramatic increase in CO_2eq_ emissions in local areas [19,60,61,62,63,64]. Emissions are further promoted by the industrial structure, which is biased toward heavy industry. Inner Mongolia is a resource-based region with economic development that heavily relies on secondary industries with high energy consumption and high pollution. Thus, industrial transformation is relatively difficult. The industrial structure should be optimized to reduce CO_2eq_ emissions in all counties of Inner Mongolia. This need was confirmed by the spatial panel econometric results for the industrial structure in this paper.

However, the effect of the industrial structure is relatively large. Thus, the unreasonable industrial structure restrains the development of a low-carbon economy in this area. Moreover, optimizing the industrial structure is conducive to energy savings and emission reductions in Inner Mongolia. The influence of such a shift in adjacent areas via spillover effects would be the same but not as significant. We attribute this phenomenon to the fact that the CO_2eq_ emission reductions achieved by optimizing the industrial structure in Inner Mongolia mainly influence local counties rather than radiating to a larger area due to regional differences, which restrains the benefits of transforming the industry. Inner Mongolia, which is currently in the process of rapid industrialization, has a large secondary industry that will continue to grow, but the emerging tertiary industry is also relatively important, and it is difficult to drastically change the corresponding industrial structure over a short period of time. Hence, the overdependence of the economy on secondary industry must be gradually reduced, and industrial development must shift from extensive, labor-intensive, and capital-intensive modes to intensive, knowledge-intensive, and technology-intensive modes. In addition, a resource-conserving and environment-friendly tertiary industry should be developed, and emerging low-carbon industries should be promoted for development.

Notably, the positive effects of economic growth and industrial structure changes will influence the spatial distribution of CO_2eq_ emissions. As shown in the spatial distribution map of CO_2eq_ emissions, zones with high CO_2eq_ emissions are primarily located in the southwestern and central areas of the province, which are situated in the “Golden Triangle”, encompassing Erdos, Baotou (Baotou is a relatively large city under the jurisdiction of Inner Mongolia and is the manufacturing and industrial center of Inner Mongolia.), and Hohhot (Hohhot is the capital of Inner Mongolia and an important central city in the northern border area of China.). These three cities are industrial cities characterized by rapid economic growth and heavy industry. The zones with low CO_2eq_ emissions are mainly located in the eastern and northern areas of the province, where economic development is lagging, and the industrial structure is dominated by agriculture and animal husbandry. The ecosystem here primarily consists of grasslands and forests. This finding indicates that CO_2eq_ emissions are highly related to economic development and the optimization of the industrial structure. Previous studies [39,65,66,67,68] also verified this result.

In addition to the factors discussed above, other factors play essential roles in promoting county-level CO_2eq_ emission reductions in Inner Mongolia. One factor is technological improvements. As the key driving force of economic growth, the impact of technological improvements (−0.807) on county-level CO_2eq_ emissions in Inner Mongolia is much higher than that for other factors. As a primary driver of production that can significantly improve the energy efficiency and promote the reduction of CO_2eq_ emissions, technical progress is a key factor involved in meeting emission reduction targets [69]. However, advances in technology will also result in rapid economic development [70] and an increased demand for energy. Under these conditions, the energy savings due to efficiency improvements will be offset by additional energy consumption due to rapid economic development, which is the so-called “rebound effect” [69]. As the spatial distance from industrial centers increases, access to technological advances will decrease. Thus, the energy efficiency in neighboring areas will not be significantly improved, but the energy demand and CO_2eq_ emissions will likely increase. Therefore, technological progress has a positive spatial spillover effect with some inhibitory results. In conclusion, Inner Mongolia should prioritize the introduction of advanced technology in high-carbon industries. At the same time, clean energy strategies should be developed to promote low-carbon economic development in all Inner Mongolian counties.

Another factor that has curbed the increase in CO_2eq_ emissions in Inner Mongolian counties is urbanization. Urbanization has had a positive impact on CO_2eq_ emissions in 84% of countries globally [15]. However, the effect of urbanization in Inner Mongolia has been the opposite. This phenomenon results from the uncertain effects of accelerated urbanization. Rapid urbanization can not only reduce energy consumption and emissions by effectively utilizing public infrastructure (e.g., public transportation) and enhancing the environmental efficiency [71,72], but it also leads to a higher energy demand and increased CO_2eq_ emissions [73,74,75,76]. Further urbanization will inevitably lead to an increased population and additional pressure on the fragile ecosystem [77]. Considering the actual situation in Inner Mongolia, where the urbanization level is relatively low, promoting urbanization will result in the effective use of local public infrastructure, reduced energy consumption, energy savings, and emission reductions [70]. Eventually, the threshold of the “urbanized economy” will be reached. However, in neighboring areas, the effect is the opposite. The utilization of public infrastructure due to urbanization does not have a positive effect on neighboring areas, but CO_2ea_ levels will likely increase in these areas due to rapid urbanization.

The improvement of government energy policies, changes in public perception regarding energy consumption, and energy efficiency improvements will together slow the CO_2eq_ emissions growth rate to a large extent [78]. Based on the results of this study, although the overall CO_2eq_ emissions in Inner Mongolia have increased annually, the growth rate of CO_2ea_ emissions in all counties has slowed. This result was co-achieved by the increased emphasis on emission reductions in recent years, the rapid economic development, and public concern for environmental quality. Thus, more attention has been given to low-carbon, energy-saving, and environmentally friendly technologies, as well as improvements in the effective utilization of energy resources. To further increase energy savings and reduce emissions, the spatially-dependent effects of CO_2ea_ emissions resulting from spatial interactions in neighboring areas must be considered. The spillover effect of CO_2eq_ emissions in Inner Mongolia from 2007 to 2012 was significant. The CO_2ea_ emissions in all counties are influenced not only by local relevant factors but also by those in neighboring areas. Consequently, all counties should determine the relevant dependence effects of CO_2ea_ emissions on energy conservation, emission reduction, and policy development strategies. Counties should strengthen their cooperation and work closely to ensure synergistic development in all regions to guarantee the effectiveness of policies and achieve mutual benefits in a win-win situation.

## 5. Conclusions

Based on the spatiotemporal evolution of CO_2__eq_ emissions in Inner Mongolian counties, this paper applied the SDM, a spatial panel econometric model, to quantitatively investigate the CO_2eq_ emissions and corresponding driving mechanism in 101 counties of Inner Mongolia. The following conclusions were obtained from the study.

CO_2eq_ emission reductions in Inner Mongolia are limited. The regional CO_2eq_ emissions increased by 7.270% annually during the study period, from 287.69 million tons in 2007 to 510.47 million tons in 2012. In addition, carbon emissions were found to vary greatly in the different counties of Inner Mongolia, and the regional CO_2eq_ emissions were shown to be highly spatially heterogeneous.

The urbanization level of counties, GDP per capita, and technological progress are the primary factors that affect CO_2eq_ emissions in Inner Mongolia. Specifically, technological progress and the urbanization level can reduce county-level CO_2eq_ emissions in Inner Mongolia, and the effect of the former (0.807) far exceeds that of the latter (0.143). Conversely, the GDP per capita and industrial structure promote CO_2eq_ emissions in Inner Mongolian counties. Each 1% positive increase in these factors will lead to increases of 0.366% and 0.012%, respectively, in CO_2eq_ emissions in local areas.

Moreover, the spatial spillover effects of CO_2eq_ emissions are not negligible. Among the factors that influence the Inner Mongolian counties, the spillover effect of technological progress is significant. The spatial spillover effect promotes an increase in CO_2eq_ emissions in neighboring areas (0.100). However, the impacts of the urbanization rate, GDP per capita, and industrial structure in the neighboring areas are not significant, and the corresponding spillover effects are negligible.

## Figures and Tables

**Table 1:** Statistics of carbon dioxide equivalent (CO_2eq_) and contributing factors in Inner Mongolian counties.

Variables	Description	Definition	Unit	Mean *	Std. Dev. *	Min *	Max *
CE	CO_2eq_ emissions	Carbon dioxide equivalent emissions produced by the industrial sector	10^4^ tons	396.51	216.74	9.17 × 10^−^^3^	7.22 × 10^3^
IS	Industrial structure	The ratio of industry sector values	%	50.52	17.60	0.01	91.85
Urban	Urbanization rate	The proportion of the urban population to the total population	%	48.32	26.91	5.45	99.70
PGDP	GDP per capita	Gross domestic product divided by the population	10^4^ CNY/per capita	5.88	6.13	6.42	3.94
VC	Output value of the construction industry	Output value of the construction industry	billion CNY	1.21	2.92	2.13 × 10^−^^3^	30.10
TP	Technical progress	GDP output per unit of energy consumption	10^4^ CNY/ton standard coal	0.86	1.43	1.65 × 10^−^^4^	10.70

Note: CNY refers to Chinese Yuan, and * represents the mean value, standard deviation, minimum value, and maximum value of the variables.

**Table 2:** Estimation results without spatial interactive effects.

Eco-spatial Weighting Matrix					Geographical Weighting Matrix			
	Pooled OLS	Spatial FE	Temporal FE	Both FEs	Pooled OLS	Spatial FE	Temporal FE	Both FEs
R^2^	0.944	0.958	0.946	0.969	0.944	0.998	0.946	0.999
σ^2^	0.225	0.007	0.2202	0.003	0.225	0.007	0.220	0.003
Lmlag	26.158 ***	132.955 ***	23.016 ***	0.400	32.469 ***	119.769 ***	33.358 ***	0.025
R_Lmlag	9.109 ***	110.550 ***	8.148 ***	5.827 **	14.627 ***	98.031 ***	15.833 ***	7.265 ***
Lmerror	60.471 ***	25.907 ***	60.459 ***	21.342 ***	58.490 ***	24.037 ***	55.346 ***	13.816 ***
R_Lmerror	43.422 ***	3.502 *	45.592 ***	26.769 ***	40.647 ***	2.299	37.821 ***	21.056 ***
LR spatially fixed joint significance (2569.947, 101, 0.000)					LR spatially fixed joint significance (2569.948, 101, 0.000)			
LR temporally fixed joint significance (472.306, 6, 0.000)					LR temporally fixed joint significance (472.306, 6, 0.000)			
Wald_spatial_lag = 34.449 ***					Wald_spatial_lag = 29.280 **			
LR_spatial_lag = 33.909 ***					LR_spatial_lag = 29.750 ***			
Wald_spatial_error = 12.714 **					Wald_spatial_error = 14.166 **			
LR_spatial_error = 14.744 ***					LR_spatial_error = 17.229 ***			

Note: ***, **, and * represent the models used in the significance test at confidence levels of 1%, 5%, and 10%, respectively. FE: fixed effect; OLS: ordinary least squares.

**Table 3:** Estimation and test results based on the spatial Durbin model (SDM) for the driving factors of CO_2eq_ emissions in Inner Mongolia.

	Eco−Spatial Weighting Matrix				Geospatial Weighting Matrix			
	Coefficient	Direct	Indirect	Total	Coefficient	Direct	Indirect	Total
*LnUrban*	−0.144 ***	−0.141 ***	0.032	−0.109 ***	−0.145 ***	−0.143 ***	0.021	−0.122 ***
*LnPGDP*	0.360 ***	0.361 ***	0.046	0.407 ***	0.365 ***	0.366 ***	0.063	0.429 ***
*LnTP*	−0.812 ***	−0.807 ***	0.083 *	−0.724 ***	−0.813 ***	−0.807 ***	0.100 **	−0.708 ***
*LnIS*	0.012 **	0.012 **	0.002	0.014 *	0.013 **	0.012 **	−0.019	−0.007 *
*LnVC*	−0.005	−0.005	0.007	0.002	−0.004	−0.005	−0.003	−0.008
W * LnUrban	0.059 *	R^2^ = 0.969 correct R^2^ = 0.799 σ^2^ = 0.004			0.047 *	R^2^= 0.999 correct R^2^=0.800 σ^2^ = 0.004		
W * LnPGDP	0.044				0.019			
W * LnTP	0.247 ***				0.239 ***			
W * LnIS	−0.001				−0.018			
W * LnVC	0.006				−0.002			
W * CE	0.221 ***				0.191 ***			

Note: ***, **, and * represent the models used in the significance test at confidence levels of 1%, 5%, and 10%, respectively.
